# Stepwise detection and evaluation reveal miR-10b and miR-222 as a remarkable prognostic pair for glioblastoma

**DOI:** 10.1038/s41388-019-0867-6

**Published:** 2019-07-09

**Authors:** Bo Sun, Xudong Zhao, Jianguang Ming, Xing Liu, Daming Liu, Chuanlu Jiang

**Affiliations:** 1 Department of Neurosurgery, the Second Affiliated Hospital of Harbin Medical University, 150086 Harbin, China; 2 College of Information and Computer Engineering, Northeast Forestry University, 150040 Harbin, China; 3 Beijing Neurosurgical Institute, 100050 Beijing, China

**Keywords:** Prognostic markers, CNS cancer

## Abstract

Despite the existence of many clinical and molecular factors reported that contribute to survival in glioblastoma, prevailing studies fell into partial or local feature selection for survival analysis. We proposed a feature selection strategy including not only joint covariate detection but also its evaluations, and performed it on miRNA expression profiles with glioblastoma. MiR-10b and miR-222 were selected as the most significant two-dimensional feature. Crucially, we integrated in vitro experiments on GBM cells and in vivo studies on a mouse model of human glioma to elucidate the synergistic effects between miR-10b and miR-222. Inhibition of miR-10b and miR-222 strongly suppress GBM cells growth, invasion, and induce apoptosis by co-targeting PTEN and leading to activation of p53 ultimately. We also demonstrated that miR-10b and miR-222 co-target BIM to induce apoptosis independent of p53 status. The results define mir-10b and mir-222 important roles in gliomagenesis and provided a reliable survival analysis strategy.

## Introduction

Glioblastoma multiforme (GBM), the highest grade of glioma with a median survival of only ~15 months, is the most common malignant primary brain tumor in adults [1]. Despite the intensive research and aggressive treatments during the past decade, prognosis for patients diagnosed with GBM remained frustrated [2]. There is still a critical need for new molecular targets and effective approaches to treat this devastating disease.

MiRNAs (miRNAs) have been found to play important roles in most biological processes, including cancer. MicroRNAs are a class of small (19–25 nucleotides), noncoding regulatory RNAs that regulate gene expression by complementary binding with the 3′-untranslated region (UTR) of target mRNAs and causing their degradation or suppressing mRNA translation [3]. Many miRNAs involved in pathogenesis of GBM and viewed as prognostic markers have been demonstrated [4, 5].

Many studies have focused on miRNA expression profiles in GBM, and provided signatures predicting survival. Anyway, most of these works fell into inappropriate statistical analyses, which made the consequent discovery unreliable. Niyazi et al. [6] used a survival cutoff value defined by the median survival days to separate long-term and short-term survivors. Besides, a variety of statistics were utilized for detection of differentially expressed individuals of miRNAs. However, stratification on patients using survival time is ill-conceived considering the right censoring of survival time. Cheng et al. [7] employed the univariate Cox regression analysis to evaluate individual miRNAs, the expression levels of which were consistent with overall survival. In fact, the usage of univariate regression satisfies the assumption that miRNAs are independent of each other. However, they kept using the regression coefficients from univariate regression as the weights of a linear combination of miRNA expression levels for calculating subsequent risk scores. On the contrary, Chen et al. [8] and Zhang et al. [9] employed the univariate Cox regression on each miRNA expressions for selection of individually significant miRNAs associated with survival time, respectively. In addition, Zhang et al. [9] adopted permutation and false discovery rate for achieving more accurate selection results. Focusing on the selected individually significant miRNAs, both of them considered the multivariate Cox regression for obtaining risk scores. Anyway, this practice violated the assumption on former usage of the univariate Cox regression. Instead, Srinivasan et al. [10] employed the multivariate Cox regression on all miRNAs, and selected those with significant regression coefficients as the miRNA candidates for further evaluation. However, this top-down strategy of miRNA selection probably may obtain redundant miRNAs. Furthermore, Niyazi et al. [11] employed a forward-selection algorithm, which was actually regarded as an incremental method, to select significant miRNAs. However, this kind of heuristic method cannot effectively prevent local optimization.

**Fig. 1:**
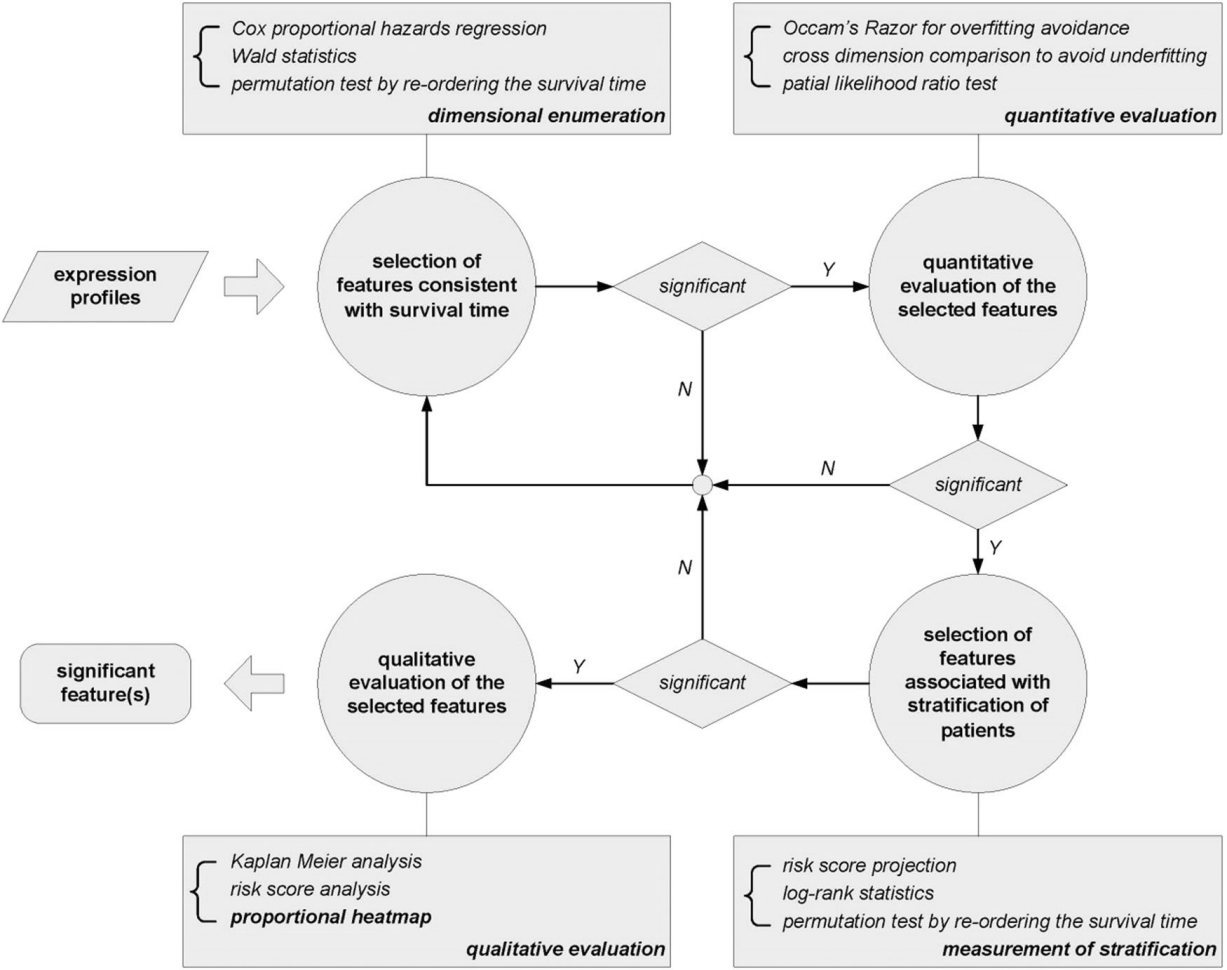
A framework indicating the strategy containing not only JCDSA-based feature selection but also the corresponding evaluations

In this paper, we developed a feature selection strategy including not only joint covariate detection for survival analysis but also the corresponding evaluation. As shown in Fig. 1, the proposed feature selection strategy contained four parts, i.e., selection of features consistent with patients’ survival time, quantitative evaluation of the selected features, selection of features associated with categories of patients with different survival risks and qualitative evaluation of the selected features. In Fig. 1, the main diagonal two parts refer to JCDSA-based feature selection, as has been expressed in our previous work [12, 13]; while, the accessory diagonal two parts correspond to its evaluation.

Moreover, we implemented the feature selection strategy on miRNA expression data (Level 3) of 548 patients with GBM publicly available at TCGA (http://cancergenome.nih.gov) and indicated miR-10b and miR-222 to be the most significant pair associated with overall survival outcomes in patients with GBM. Crucially, we identified that miR-10b and miR-222 promote GBM tumorigenesis by collectively targeting phosphatase and tensin homolog (PTEN) which activates the p53 pathway by suppressing mouse double minute 2 protein (MDM2). Besides, we further demonstrated miR-10b and miR-222 co-target Bcl2l11 (BIM) that induces apoptosis of GBM cells without p53 activation. In addition, inhibition of miR-10b and miR-222 in model mice with intracranial glioma resulted in significant reduction of tumor growth. In summary, our experimental results demonstrated a reliable survival analysis strategy, and suggested a potential new therapeutic approach in the treatment of GBM.

## Results

### Selection of features consistent with patients’ survival time

MiRNA expression data (Level 3) of 548 patients with GBM downloaded from TCGA was processed. There are many regression models for right censoring [14], which refers to the phenomenon that patients with cancer still survive till after the nearest follow-up time. Following the assumption that censoring is independent of survival time, we utilized Cox proportional hazards regression [15] to select features consistent with patients’ survival time for simplicity. Features were from one dimension to higher dimension. In other words, we enumerated on individual, double and triple combinations of miRNA expressions to search features in that dimension, which were thought to be consistent with patients’ survival time. Permutations by re-ordering the survival time for 10,000 rounds were made. Moreover, significant features, each component of which kept a small *p* value (*p* ≤ 0.001), were selected as shown in Supplementary Table S1, Table S2, and Table S3. As a result, 9 individuals, 26 pairs, and 68 triples of miRNAs were selected. More details about how the *p-*values were obtained could be seen in the method part of our previous work [12, 13].

### Quantitative evaluation of the selected features

After selecting features consistent with survival time, significant individuals, pairs or triples were sent for quantitative evaluation. Dimensional enumeration cannot go on ceaselessly. Therefore, Occam’s Razor [16] was considered in order to decide when to terminate feature enumeration. After making a careful comparison among the *p*-values from one-dimensional, two-dimensional, and three-dimensional features, we decided to choose the 26 pairs of miRNA rather than 68 triples for further screening on account of the overfitting avoidance.

Then, we made a cross dimension comparison for under-fitting avoidance. Taking one selected pair miR-10b and miR-222 as an example, it was found that not miR-10b but miR-222 was individually significant (see Supplementary Table S1). Thus, we have to discuss whether miR-10b is still needed or not. Based on the Cox hazards assumption [15], hazard ratio is strongly associated with Cox regression coefficients. That is, whether miR-10b is necessary or not depends on the coefficient change of miR-222. Table 1 integrated Cox regression coefficient with hazard ratio (HR) between different patients’ risk groups and 95% confidence interval estimate (CIE), together with *p*-values corresponding to survival time. We calculated the change ratio of Cox regression coefficients corresponding to miR-222, as was expressed a$\Delta \hat \beta$ = (0.3061−0.2456)/0.3061 = 0.1976. The change ratio was considered large, which demonstrates the effectiveness of the pair miR-222 and miR-10b rather than only miR-222.

**Table 1:** Comparisons between univariable and multivariable model

Model	Variable	Coefficient	HR	95% CIE	*P-*value
Univariable	miR-222	0.2456	1.2783	[1.1848, 1.3793]	0.0001
	miR-10b	0.0360	1.0367	[0.9701, 1.1078]	0.2824
Multivariable^a^	miR-222	0.3061	1.3581	[1.2493, 1.4766]	0.0001
	miR-10b	0.1412	1.1517	[1.0675, 1.2425]	0.0004

^a^*ll*($$\hat \beta$$) = −2.354 × 10^3^, *ll*(0) = −2.3815 × 10^3^, *T* ~ *χ*^2^ (2), *p* = 1.14 × 10^−12^

In order to check whether each selected pair was of multivariate significance or not, we made partial likelihood ratio test on each selected miRNA pair. Taking the selected pair miR-222 and miR-10b as an example, the log partial likelihood function *║*, the statistic *T*, and the corresponding *p*-value were listed in Table 1. More details could be seen in “Methods section”.

### Selection of features associated with stratifications of patients

After quantitative evaluation, we moved on to selection of features associated with stratification of patients. In practice, clinical patients are commonly classified into low-risk and high-risk group, which conforms to therapists’ decision-making process. We employed log-rank test to assess the statistical significance of differences between the two risk groups. A risk score on each sample was obtained using the linear combination on the components’ expression levels of each selected miRNA candidate weighted by Cox regression coefficients. For simplicity, a cutoff threshold was derived from the median risk score. Thereafter, patients were stratified using their risk scores. According to log-rank test, we proposed a permutation test and calculated the corresponding *p*-value. For each of the 26 significant miRNA pair, *p*-value corresponding to log-rank test was calculated. Those with their *p*-values < 0.001 were thought to be discriminative and selected. Thus, six significant miRNA pairs, which were not only consistent with patients’ survival time but also associated with differentiation between patients’ risk groups, were further selected as listed in Supplementary Table S4. Furthermore, we more stringently controlled the threshold of *p*-value to be 0.0005, and ultimately selected the pair miR-222 and miR-10b for further analysis. More details about how the *p*-values were obtained could be seen in the method part of our previous work [12, 13].

### Qualitative evaluation of the selected features

**Fig. 2:**
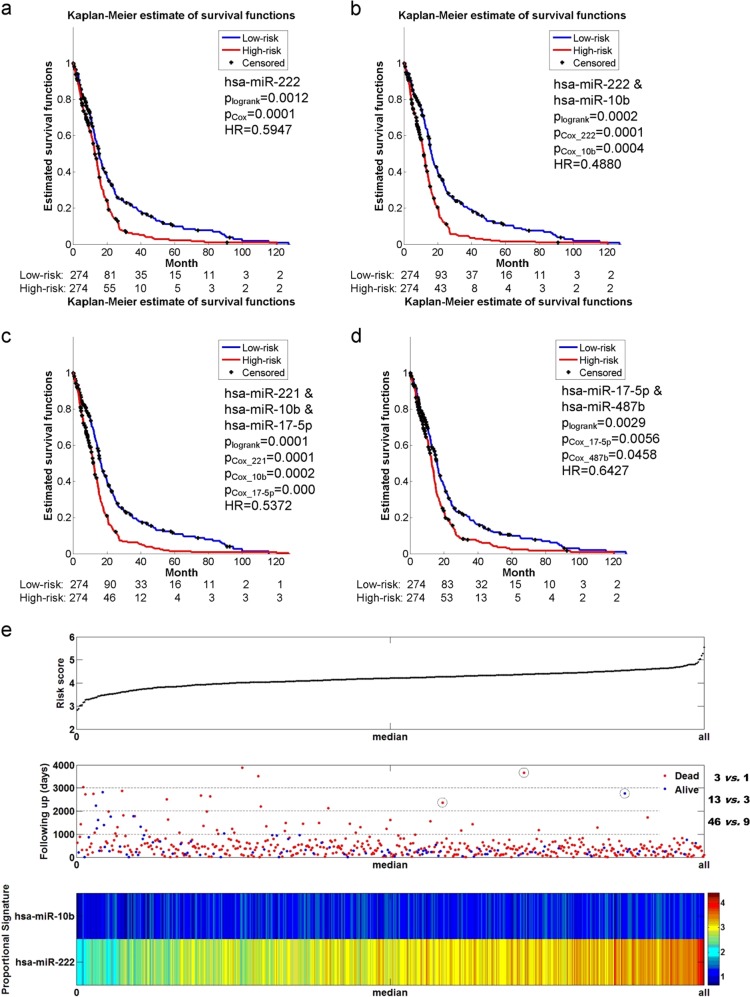
Qualitative evaluation of the selected features. **a** Kaplan–Meier plots using the most individually significant miRNA, i.e. miR-222. **b** Kaplan–Meier plots using the most significant pair, i.e., miR-222 and miR-10b. **c** Kaplan–Meier plots using the most significant triple, i.e., miR-221, miR-10b, and miR-17-5p. **d** Kaplan–Meier plots that combine individually significant miRNAs, i.e. miR-17-5p and miR-487b. **e** Risk-score analysis including a plot of each patient’s risk score value, a scatterplot of each patient’s following-up day and a proportional heatmap that indicates the weighted expression levels of miR-222 and miR-10b

Other than quantitative analyses, clinicians prefer qualitative results in practice. Kaplan–Meier survival analysis is commonly used to show differences between the two patients’ risk groups. We listed four Kaplan–Meier plots for visualization analysis, as illustrated in Fig. 2. The first three panels, i.e., Fig. 2a–c, indicated survival differences using the most quantitatively significant miRNA individual, pair and triple, respectively. After making a careful comparison, we demonstrated that the selected miR-222 and miR-10b together kept the best performance. In addition, we particularized all possible combinations of individual significant miRNAs and showed the Kaplan–Meier plot corresponding to the combination with the best performance, as shown in Fig. 2d. Comparisons between Fig. 2b, d indicated that the significant pair which kept best performance was not composed of significant individuals.

Moreover, we depicted a proportional risk-score analysis to show the effectiveness of our selected significant miRNA pair, as illustrated in Fig. 2e. Patients were re-ordered from the smallest to the biggest risk score. The corresponding follow-up days of patients and the weighted expression levels of the two miRNAs were listed. The median risk score was regarded as a cutoff value, which was utilized to divide patients into a low-risk group at the left side and a high-risk group at the right side. It could be seen in the scatterplot of each patient’s following-up day that the low-risk group kept more patients with longer survival days than those in the high-risk group. In other words, miR-222 and miR-10b could stratify patients’ survival risks. Corresponding to the risk scores of patients, a proportional heatmap representing the weighted expression levels of the two miRNAs derived from Cox regression coefficients was shown. It could be seen that miR-222 was an over-expressed variable for the risk of death. As to miR-10b, it was a little hard to find any obvious change on expression levels between the high-risk and low-risk group. In assistance of the estimated HR and the 95% CIE listed in Table 1, we confirmed that miR-10b was an over-expressed variable. It could be seen that the 95% CIE of estimated HR included one, which indicated that there might be no apparent difference between the high-risk and low-risk group. However, it excluded one in the corresponding multivariable Cox regression model, which verified that miR-10b together with miR-222 was over-expressed for the risk of death.

### MiR-10b and miR-222 regulate glioma cells proliferation and invasiveness via p53 pathway

**Fig. 3:**
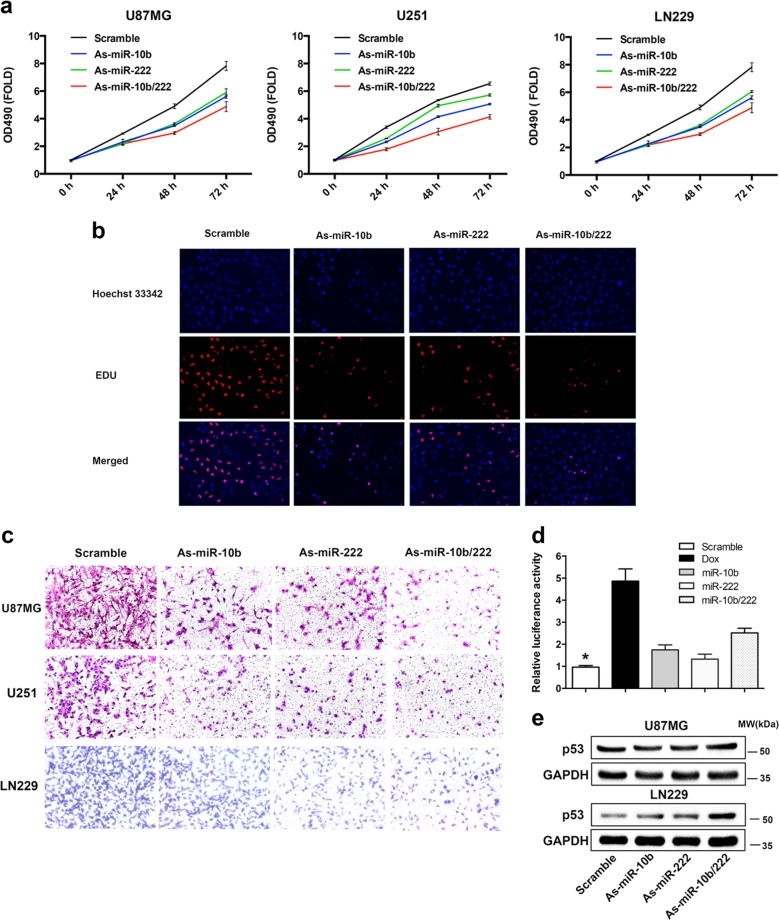
Inhibition of miR-10b and miR-222 suppressed the GBM cells proliferation and invasiveness by activation of p53. **a** LN229, U87MG, and U251 cells were transfected As-miR-10b or/and As-miR-222 and harvested after 24, 48, and 72 h. Cell viability was analyzed by CCK‐8 assay. **b** Representative results of the EdU proliferation assay of LN229. All cells were transfected with miRNA inhibitors and labeled with EdU (red), Hoechst 33342 (blue). **c** Representative images of Transwell assays of cells after transfection with miRNA inhibitor. **d** LN229 cells were co-transfected with pGL4.38 [luc2P/p53 RE/Hygro] and pGL4.74 [hRluc/TK] vector plasmids. 24 h after transfection, the cells were treated with miR-Scr, As-miR-10b, As-miR-222, and As-miR-10b/222 for 24 h. Cells were only stimulation with doxorubicin (5 mM) for 18 h as positive control. The data were represented as mean ± s.d. (*n* = 3), *p < 0.01 versus scramble group. **e** Western blot analysis showed the level of p53 after inhibition of miR-10b and miR-222 in LN229 and U87MG. GAPDH was used as an internal control. The RT-PCR results for *TP53* mRNA in LN229 and U87MG were show in Supplementary Fig. S2

To identify the tumorigenic roles of miR-10b and miR-222 in GBM, we determined whether downregulation of these miRNAs would have effects on the viability and invasiveness of GBM cells. As shown in Fig. 3a, striking reduce of the growth was observed in all three cell lines by transfecting miRNA inhibitors. Similarly, inhibition of miR-10b and miR-222 also decreased the cell proliferation in all the cell lines by the EdU proliferation assay (Fig. 3b, Supplementary Fig. S1). Moreover, the transwell assay revealed that miR-10b and miR-222 regulated the invasion capacity of GBM cells compared with negative control (Fig. 3c). To further elucidate the underlying mechanism or potential synergistic effects between miR-10b and miR-222, we utilized DIANA miRPath [17] which provided high quality experimentally validated miRNA/gene interactions to identify the KEGG pathway that was targeted by both miR-10b and miR-222. Consequently, Cell cycle and p53 signaling were ranked among the four pathways likely to be co-regulated by miR-10b and miR-222 (Supplementary Table S5). To verify this conjecture, p53 transcriptional activity was measured by the luciferase assay. We showed that inhibition of miR-10b and miR-222 significantly enhanced the p53 transcriptional activity relative to the control in LN229 (Fig. 3d). It was noteworthy that the protein level of p53 was increased in LN229, but there was no change in U87MG(Fig. 3e). Besides, neither LN229 or U87MG showed any change in mRNA level of p53 (Supplementary Fig. S2), even if both cell lines contained functional p53 [18]. Then we found that both miR-10b and miR-222 could not bind p53 mRNA by using the miRWalk target prediction program [19]. Based on these results, we speculated that miR-10b and miR-222 may influence p53 transcriptional activity and result in different presentations between LN229 and U87MG cell lines by other mechanism without directly targeting p53.

### Downregulation of miR-10b and miR-222 results in p53 activation by suppressing MDM2

**Fig. 4:**
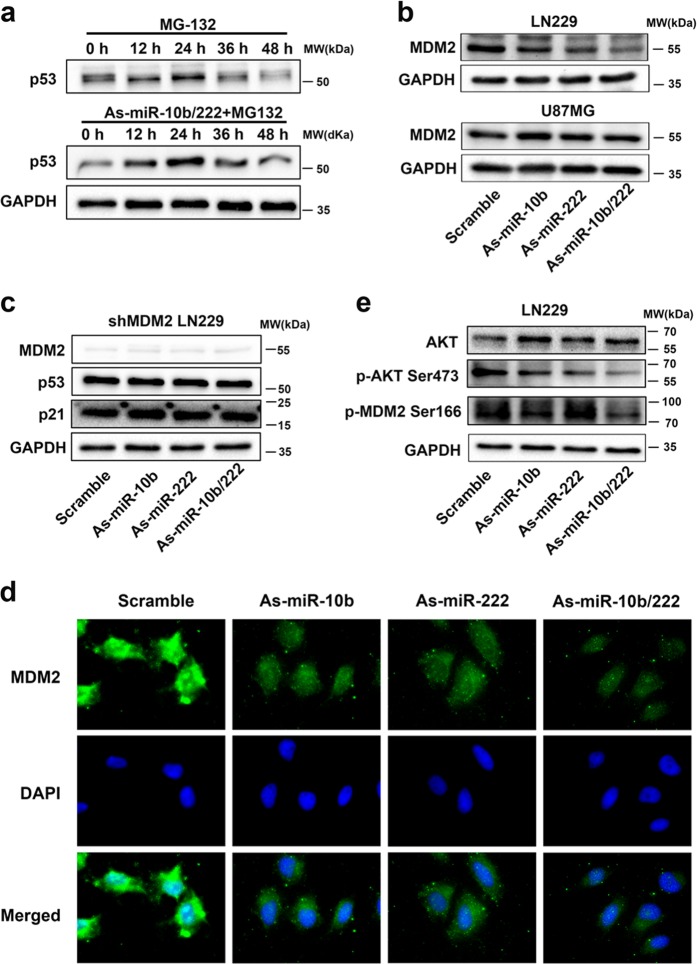
Downregulation of miR-10b and miR-222 suppressed the express of MDM2. **a** LN229 cells were treated with MG132 (10 μmol/L) alone for 6 h and harvested different hours after treatment as control. Cells were transfected with As-miR-10b/222 for 24 h and together with MG132 (10 μmol/L) treatment as compared. The difference levels of p53 between the two groups were analysis by WB. **b** Western blot analysis showed the levels of MDM2 after inhibition of miR-10b and miR-222 in LN229 and U87MG. **c** The level of p53 was tested by WB, after inhibition of miR-10b and miR-222 in LN229 with MDM2 stable knockdown. **d** Representative images of IF using anti-MDM2 antibody shows the subcellular distribution of MDM2 protein. **e** Western blot analysis showed the levels of MDM2 Ser166 and AKT Ser473 in LN229 cells that transfected with miRNA inhibitors as indicated. The results have been correct with the downregulation of total MDM2

We have revealed that the increased protein level of p53 was not due to the enhance of transcription of p53. Therefore, we speculated that inhibition of miR-10b and miR-222 increased the level of p53 protein by post-translational modification, which stabilized and inhibited degradation of the p53 protein. To test this hypothesis, LN229 cells were transfected with miR-10b/-222 inhibitors combined with the treatment of MG132, a potent inhibitor of proteasome, and treated with MG132 alone as control group. The western blot (WB) results suggested that inhibition of miR-10b and miR-222 resulted in the increased protein level of p53 by inhibition of proteolysis (Fig. 4a). Mouse double minute 2 protein (MDM2) is known as a key p53 inhibitor which can directly bind p53, promote its ubiquitination and shuttle it to cytoplasm, where it is subsequently degraded by the proteasome [20]. To test whether miR-10b and miR-222 could modulate the expression of MDM2, we tested the protein levels of MDM2 in LN229 and U87MG cells that was treated with the inhibitor of miR-10b and miR-222. As expected, the protein levels of MDM2 were markedly reduced in LN229 and still did not change in U87MG (Fig. 4b). To further testify the accumulation of p53 due to the inhibition of MDM2 by miR-10b and miR-222, LN229 cells with stable MDM2 knockdown were established. The knockdown efficiency of MDM2 on LN229 was tested by WB (Supplementary Fig. S3). The inhibition of miR-10/222 in MDM2 knockdown of LN229 cells did not change the p53 levels (Fig. 4c). These results confirmed that the accumulation of p53 in LN229 that treated with As-miR-10b and As-miR-222 was mediated by MDM2. The co-immunoprecipitation (Co-IP) assay in LN229 was performed to check the binding status of MDM2-p53. The results just consistent with the suppression of MDM2 and the less MDM2-p53 binding (Supplementary Fig. S4). The ability of MDM2 to shuttle between the nucleus and the cytoplasm is required to bind p53 and depends on the phosphorylation of MDM2 by phosphatidylinositol 3′-kinase (PI3K)/AKT pathway [21]. Hence, we utilized immune-fluorescence (IF) analysis to further confirm the expression and nucleus shuttle of MDM2 and the western blot for the analysis of PI3K/AKT activation. The IF analysis indicates that the expression of MDM2 was significantly reduced and the MDM2 shuttling defect is not apparent (Fig. 4d). However, the levels of MDM2 Ser166 and AKT Ser473 were both reduced in LN229 tested by WB (Fig. 4e). Base on all above results, we concluded that the target genes of miR-10b and miR-222 in GBM cells could downregulate the expression of MDM2 and inhibit AKT activation to phosphorylate MDM2 as well.

### MiR-10b and miR-222 directly target PTEN in GBM cells

PTEN is regarded as a well-known antagonizer for PI3K/AKT pathway by negatively regulating AKT activation through PIP3 dephosphorylation [22]. Besides, PTEN also inhibits MDM2 by suppressing its P1 promoter, blocking MDM2 nuclear translocation and destabilizing the MDM2 protein [23]. These findings led us to test whether miR-10b and miR-222 could co-target PTEN that accounts for all above presentations. Meanwhile, analysis of mir-10b and mir-222 predicted targets by miRWalk revealed that PTEN as a predicted target gene of the two miRNAs [19].

**Fig. 5:**
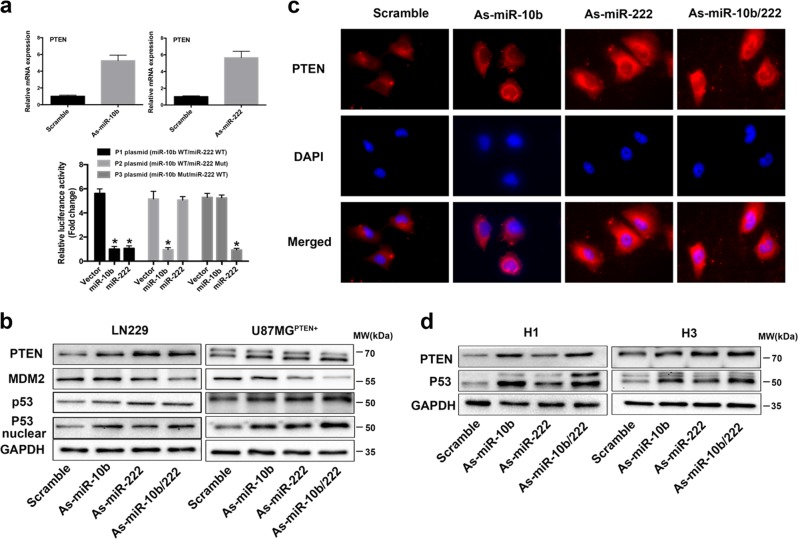
MiR-10b and miR-222 direct target PTEN in GBM. **a** Luciferase reporter assays in glioma cells after co-transfection of cells with P1, P2, and P3 plasmids and miRNA mimics. The data was represented as mean ± s.d. (*n* = 3), *p < 0.01 versus vector group for each plasmid. Inhibition of miR-10b and miR-222 increased PTEN expression test by RT-PCR. **b** Western blot analysis for PTEN, MDM2, and p53 in LN229 and U87MG^PTEN+^. All cells were harvested at 48 h after transfection with miR-Scr or As-miR-10b/222 as previously indicated. **c** Representative images of IF using anti-PTEN antibody showed the level and subcellular distribution of PTEN protein in LN229. **d** Western blot analysis of PTEN and p53 in glioma derived primary cell cultures after transfection with As-miR-10b/222 as indicated. GAPDH was used as loading control

We constructed dual-luciferase reporter plasmids containing either wild-type or mutated 3′UTRs of PTEN for miR-10 or miR-222 (miR-222^wt^/miR-10b^wt^, miR-222^wt^/miR-10b^mut^, miR-222^mut^/miR-10b^wt^). The seed-bind sequence and mutations designed were indicated in Supplementary Fig. S5. The luciferase activity results verified that miR-10b and miR-222 could co-target PTEN. Meanwhile, downregulation of miR-10b and miR-222 also increased PTEN expression demonstrated by RT-PCR (Fig. 5a). Interestingly, only LN229 bears wild-type PTEN, both U87MG and U251 lost functional PTEN expression. That’s probably the reason why U87MG and LN229 represented so many differences in WB results. To clearly demonstrate the mechanism, we transfected both U87MG and U251 with wild-type PTEN plasmid (Supplementary Fig. S6) and tested PTEN/MDM2/p53 protein levels (Fig. 5b). Finally, it was all confirmed in both LN229 and U87^*PTEN+*^ cells that inhibition of miR-10b and miR-222 increased PTEN level that suppressed MDM2 and resulted in accumulation protein of p53. The IF analysis of PTEN was in accord with the WB results and just opposite of MDM2 (Fig. 5c). The data about U251^*PTEN+*^ was not shown due to the mutant of p53. As the highly molecular heterogeneity of GBM from different individuals and the genetically unstable of culture cell lines, we confirmed the roles of miR-10b and miR-222 in PTEN/MDM2/p53 interactions by using two primary GBM patients derived cells (H1 and H3) (Fig. 5d). In summary, miR-10b and miR-222 promote glioblastoma cell growth by targeting PTEN to disturb p53 function.

### Inhibition of miR-10b and miR-222 induces cell cycle arrest and apoptosis by PTEN/p53-dependent and -independent ways

**Fig. 6:**
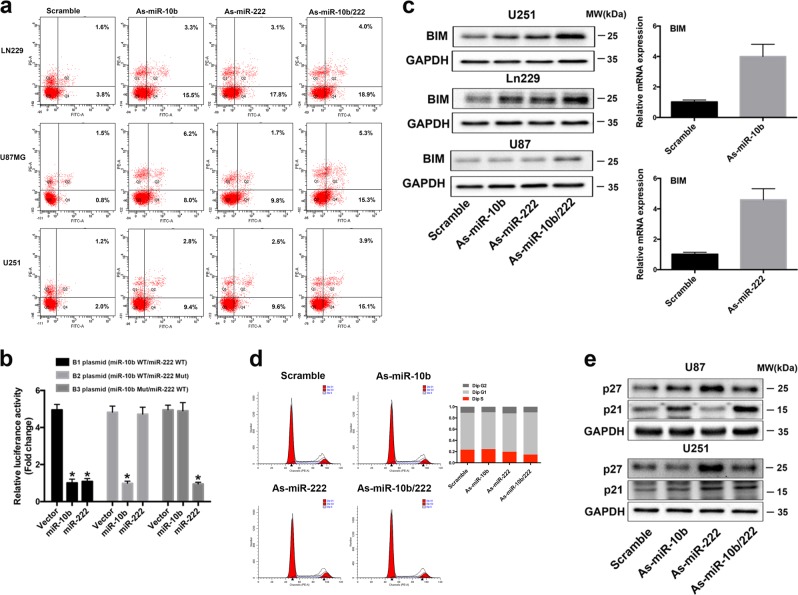
Inhibition of miR-10b and miR-222 induced cell cycle arrest and apoptosis of GBM cells, and BIM was co-targeted by miR-10b and miR-222. **a** The LN229, U87MG, and U251 cells were transfected with indicated miRNA inhibitors for 72 h, and the promotion of apoptosis was measured by AnnexinV‐FITC/PI double staining. **b** Luciferase reporter assays in glioma cells after co-transfection of cells with B1, B2, and B3 plasmids and miRNA mimics. The data were represented as mean ± s.d. (*n* = 3), *p < 0.01 versus vector group for each plasmid. **c** Western blot and RT-PCR analysis for BIM in LN229, U251, and U87MG. **d** Cell cycle analysis of miR-Scr and As-miR-10b/222 transfected cells in LN229, and overview of the cell cycle. **e** Western blot analysis for p21 and p27 in U251 and U87MG after transfection as indicated

The activation of p53 facilitates the cellular response to genotoxic stress, and initiates DNA repair, cell-cycle arrest, and importantly, apoptosis [24]. We first examined whether inhibition of miR-10b and miR-222 could induce apoptosis in three cell lines bearing different PTEN/p53 status (LN229^*pten+/p53+*^, U87MG^*pten–/p53+*^, and U251^*pten−/p53−*^) [25, 26]. The results revealed that inhibition of miR-10b and miR-222 induced the apoptosis in all three cell lines (Fig. 6a), and the expressions of several principle genes regulated the apoptosis (including BAX, PUMA, and caspase 3) were also elevated in all cell lines (Supplementary Fig. S7). Meanwhile, our previous study demonstrated that miR-222 targeted BCL2 binding component 3 (PUMA) to induce apoptosis independent of p53 status [27]. Accordingly, we hypothesized that inhibition of miR-10b and miR-222 to induce apoptosis in GBM cells may exist a mechanism independent of p53 activation.

To test this hypothesis, we focused on the targets of miR-10b and miR-222 that could modulate apoptosis. Thereafter, we found BIM/BCL2L11 had putative binding sites for both miR-10b and miR-222 by using the miRWalk target prediction program [19]. BIM, a proapoptotic BH3-only protein of the Bcl-2 family, is an essential initiator of apoptosis [28]. To validate the direct binding and targeting for miR-10b and miR-222, we also constructed dual-luciferase reporter plasmids containing either wild-type or mutated 3′UTRs of BIM (miR-222^wt^/miR-10b^wt^, miR-222^wt^/miR-10b^mut^, and miR-222^mut^/miR-10b^wt^). The seed-bind sequence and mutations were designed and indicated in Supplementary Fig. S8. As expected, the luciferase activity showed significant differences among the three reporters that transfected with miR-10b or miR-222 mimics (Fig. 6b). In addition, the high expression of mRNA and protein of BIM were also detected by WB and RT-PCR in all cell lines (Fig. 6c). These data confirmed that miR-10b and miR-222 also directly modulated BIM expression by binding to the 3′UTR to induce apoptosis by p53 independent mode.

The flow cytometry analysis showed that LN229 exhibited cell cycle arrest at G1 phase after inhibition of miR-10b and miR-222 (Fig. 6d). However, without activation of p53, U87MG and U251 similarly accumulated in G1 phase in both miR-10b/222 inhibition group (Supplementary Fig. S9). MiR-222 has been validated to target the 3′ untranslated regions of p27/Kip1 [29]. In addition, silencing of miR-10b caused de-repression of CDKN1A/p21 by direct targeting its 3′ UTR and led to reduction of glioma cell growth [30]. p27^Kip1^ and p21^Cip1^ are thought to suppress tumor growth and prevent cell cycle progression by inhibiting Cdk2-cyclin E/A kinases [31]. In according to these previous studies, we confirmed the levels of p21 and p27 were all increased in GBM cells that transfected with miR-10b/-222 inhibitors (Fig. 6e). These data might indicate how inhibition of miR-10b and miR-222 could induce cell cycle arrest without activation of p53 in U87MG and U251 cells.

### Anti-miR-10b/miR-222 attenuate growth of GBM cells in vivo

**Fig. 7:**
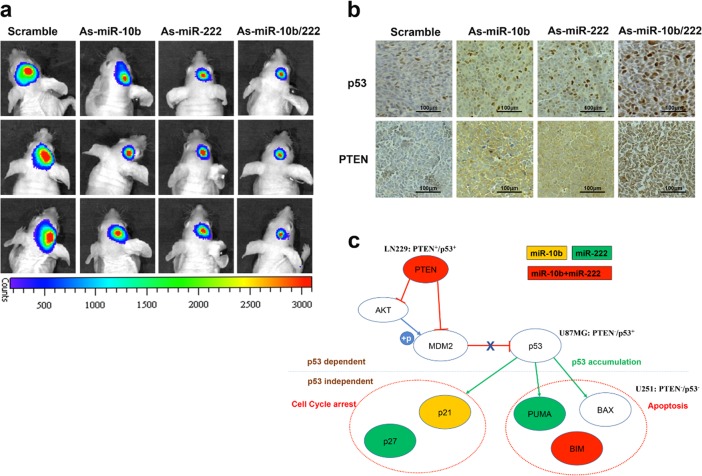
Downregulation of miR-10b and miR-222 inhibited tumor growth in vivo. **a** Representative luminescence imaging for As-miR-10b/222 transfected LN229-luc tumors versus scramble-treated controls (images were taken in the third week after treatment). **b** PTEN and p53 expression after transfecting in tumor sections following IHC analysis (*the bars represent 100 μm). **c** Schematic representation of the pattern for miR-10b and miR-222 participated in tumorigenicity of GBM. Model summarizes the contribution of miR-10b and miR-222 to target PTEN and BIM to suppress p53 pathway. PUMA, p21 and p27 were also involved in the regulation of tumor by miR-10b/222. MiR-10b target gene was shown in yellow. MiR-222 target genes were shown in green. Co-target genes by miR-10b and miR-222 were shown in red

To verify the physiological evidence of miR-10b and miR-222 regulation to glioblastoma cell growth, we further assessed the antitumor efficacy of As-miR-10b/miR-222 in vivo. We adopted nude mice bearing intracranial glioma cells (LN229-Luc) and divided into four group (*n* = 4/group, that were separately transfected with As-miR-10b, As-miR-222, As-miR-10b/222, and scrambled as negative control). We assessed tumor growth of the four mice groups by bioluminescence imaging every week. All the miRNA inhibitors transfected groups showed considerable reduction of tumor compared with the scrambled group (Fig. 7a). Immunohistochemistry (IHC) analysis confirmed that inhibition of miR-10b and miR-222 increased PTEN and p53 levels in tumor, compared with the control group (Fig. 7b). Altogether, these results indicated that downregulation of miR-10b and miR-222 suppressed GBM tumor growth by targeting PTEN/p53 similarly in vivo.

## Discussion

We have presented a method named as joint covariate detection [12] for selection of features by bottom-up enumeration of variables, and have provided a tool named as JCDSA, i.e., a joint covariate detection tool for survival analysis [13]. Joint covariate detection resolved the problem of partial or local feature selection by variable enumeration. It has been revealed that dimensional features considered to be significant may not be composed of variables individually significant by comparisons within joint covariate detection on different dimensions or between joint covariate detection and other prevailing model (e.g., random survival forest [32]) for feature selection on simulation data, which also helps to demonstrate that joint covariate detection can select true features rather than false positives [13]. However, it lacks evaluation steps for feature confirmation. Therefore, we proposed evaluations of JCDSA-based feature selection, which together with joint covariate detection formed a bottom-up feature selection strategy as shown in Fig. 1.

Besides, our contributions are several folds. First, quantitative evaluation of the selected features consistent with survival time was considered. In order to decide when to terminate feature enumeration, Occam’s Razor and cross dimension comparison were utilized for overfitting and under-fitting avoidance, respectively. Second, qualitative evaluation of the selected features associated with stratification of patients was also made including a proportional heatmap. As was firstly provided, the proportional heatmap considers the correlation of each component of the selected feature, which is totally different from the traditional exhibition of expression values of significant individuals. In that way, potential variable (e.g., miR-10b) might probably submerge on account of the significant individuals (e.g. miR-222) with absolutely large expression values. The above two points have been visually shown in Fig. 2. Last but not least, we divided joint covariate detection into sequential steps, i.e., selection of features consistent with survival time and selection of features associated with stratification of patients, which significantly reduced the processing time and made possible the accomplishment of triple feature enumeration. Using High-performance computing (HPC) which kept 960 cores in 40 nodes, we became the first to fulfill triple feature enumeration on miRNA expression data (Level 3) of 548 patients with GBM downloaded from TCGA spending over 48 days.

Mir-10b and miR-222 have been well demonstrated as important onco-miRNAs that promote tumorigenesis and maintain malignancy in GBM cells [30]. Notably, the high expression of miR-10b and miR-222 were both significantly associated with poor survival in glioma patients [30, 33, 34]. In our study, we implemented a strategy containing not only JCDSA-based feature selection but also the corresponding evaluations and found out miR-10b and miR-222 as the most significant pair associated with overall survival in patients with GBM. To testify the result and clarify the molecular mechanism that these miRNAs involved, we first focused on the p53 pathway by bioinformatics analysis (i.e., DIANA [28]). Then we confirmed that the activation of p53 due to the suppression of MDM2 expression and the blocking of phosphorylation MDM2 by p-AKT. Ultimately, it was confirmed that these cellular responses were mediated by increased expression of PTEN, which is co-targeted by miR-10b and miR-222. MiR-222 has been reported that directly target PTEN in aggressive non-small cell lung cancer, hepatocarcinoma, and gastric carcinoma [35, 36]. Meanwhile, miR-10b was found to regulate the self-renewal of the breast cancer stem cell phenotype by targeting PTEN [37]. But the interaction between PTEN and p53 was not explored in any miR-222 or miR-10b function studies. We demonstrated for the first time that miR-10b and miR-222 co-target PTEN in GBM and elucidated the activation of p53 by PTEN/MDM2 interaction. *PTEN* and *TP53* are the two key tumor suppressor genes. Although they are functionally distinct and involved in different pathway, reciprocal cooperation has been well proposed, as PTEN is thought to regulate p53 stability by suppressing MDM2, and p53 to enhance PTEN transcription [38]. Meanwhile, *PTEN* and *TP53* are also the most commonly mutated genes in human cancer including GBM [39]. Numerous investigations have shown that the inactivation of both genes is required for gliomagenesis [40]. The significance of our study on miR-10b and miR-222 is that it further reveals the mechanism of PTEN and p53 inactivation in gliomas.

The apoptosis in U87MG and U251 cells induced by inhibition of miR-10b and miR-222 attracted our attentions to the mechanism independent of *PTEN/TP53* status. Resistance to apoptosis is a major obstacle in GBM therapy [41]. We further identified the proapoptotic molecule BIM as a common target of miR-10b and miR-222 in GBM. BIM is localized to the mitochondria where it initiates the mitochondrial cell death pathway by directly activating Bax/Bak-dependent apoptosis [5]. Mir-10b has been demonstrated to control the growth of gliomas by targeting BIM [30]. However, miR-222 was only reported to target BIM in PC12 cells [42]. BIM is downregulated in 29% of GBM cases based on TCGA analysis [5]. The further study of miR-10b and miR-222 in cell cycle arrest and apoptosis suggested their roles in tumorigenicity independent of PTEN/p53 interaction and probably explained the reason for the downregulation of BIM in such GBM patients. In summary, our results demonstrated the central roles of miR-10b and miR-222 in well-known tumor suppressor genes’ network and novel apoptosis inducement as schematically illustrated in Fig. 7c. Inhibition of miR-10b and miR-222 in model mice with intracranial glioma resulted in significant reduction of tumor growth. The study therefore represents combination of miR-10b and miR-222 could be a promising therapeutic strategy for GBM patient.

## Methods

In order to make an assurance on selecting significant miRNA pairs other than individual miRNAs, a comparison of Cox proportional hazard regression coefficients was made between multivariable regression on the significant pair and univariable regression on the corresponding individually significant variable, which was a part of that significant pair. Note that the established model was a multivariable one. Thus, partial likelihood ratio test [14] denoted as *T* was calculated as follows,

1 $$T = 2\left[ {ll\left( {{\hat{\mathbf \beta }}} \right) - ll\left( {\mathbf{0}} \right)} \right],$$

where *ll* denotes the log partial likelihood function. *T* follows a *χ*^2^ distribution with *k* degrees of freedom. *k* denotes the feature dimension. If its corresponding *p*-value is significance together with significant *p*-values in each component (see the method part of our previous work [12]), the selected features are quantitatively confirmed.

### Oligonucleotides, reagents treatment

The miR-10b and miR-222 inhibitors (As-miR-10b, As-miR-222), mimics and corresponding control RNA were obtained from GeneP Pharma (Suzhou, china; see Supplementary Table S6 for detailed sequences). Cells were transfected with mimics or inhibitor of miRNAs using Lipofectamine 2000 (Invitrogen; LS11668019) according to the manufacturer’s instructions. MG132(S2619) and Doxorubicin(S1208) were obtained from Selleck Chemical. We purchased G418(E859) from Amresco.

### Plasmids and short hairpin RNA transfection

The PTEN (NM_000314) plasmid and plain vector (CMV-MCS-EGFP-SV40-Neomycin) were purchased and constructed from Genechem Company (Shanghai, China). The shMDM2-Plko.1-Puro plasmids and Control short hairpin RNA (shRNA) vector were constructed from Ribobio Company. The MDM2 shRNA1, shRNA2, shRNA3, and shRNA4 sequence were listed in Supplementary Table S7. The plasmid profiles were show in Supplementary Fig. S10. Stable cell line for the expression of PTEN or knockdown MDM2 were selected using 0.8 mg/mL G418 (Amresco) for 2 weeks and then cultured in 10% FBS with 0.4 mg/mL G418. G418-resistant colonies were cloned or pooled for analysis.

### Cell lines culture

The human glioma cell lines, U87MG, U251, and LN229, were purchased from Chinese Academy of Sciences Cell Bank. To avoid cross-contamination, all cell lines have been confirmed by short tandem repeat (STR) tests. We also test for mycoplasma contamination. The LN229 cells were cultured in Dulbecco’s modified Eagle’s medium (DMEM)/F12 (Corning) supplemented with 10% fetal bovine serum (Gibco) and 1% antibiotic (Sigma). The U87 and U251 cells were maintained in Dulbecco’s modified Eagle’s medium (Gibco) supplemented with 10% fetal bovine serum (Gibco). Patient derived glioma cells were grown in DMEM (F-12) supplemented with B-27 (Thermo Fisher Scientific; 17504044), 20 ng/ml basic fibroblast growth factor (Peprotech; 100-18B) and 20 ng/ml epidermal growth factor (Peprotech; AF-100-15). All cells were incubated at 37 °C in a 5% CO_2_ atmosphere.

### Cell proliferation assay

The glioma cells growth treated by As-miR-10b, As-miR-222, and As-miR-10b/222 were evaluated using the Cell counting kit-8 (CCK-8) (Dojindo, CK04) assay according to the manufacturer’s instructions. In brief, cells were seeded at density of 3000–5000 cells/well in a 96-well plate and incubated overnight. After transfections for 24, 48, and 72 h, CCK-8 (10 μl, 10%) was added to each well once every hour before incubation ended. Then its absorbance at 450 nm was measured by a microplate reader (IMARK). All experiments were repeated in triplicate.

### EdU proliferation analysis

Cell-Light 5-Ethynyl-2′-deoxyuridine (EdU) Cell Proliferation Kit (RiboBio; C10310) was used to investigate the proliferation of GBM cells according to the manufacturer’s instructions. In brief, all cells were seeded in a 96-well plate, and the medium was replaced with 100 μL of 10 μM EdU medium in each well. The cells were then incubated for 2 h and fixed with 4% paraformaldehyde. The formaldehyde was neutralized with 2 mg/mL glycine solution, after which the cells were subjected to 0.5% Triton X-100 permeabilization. Then, the cells were stained with Apollo® reaction cocktail and incubated for 30 mins. The cells were subsequently counterstained with Hoechst 33342 for 20 mins and imaged via fluorescence microscopy (Nikon, Japan).

### The transwell invasion assay

Invasion assays were performed in a 24-well Transwell chamber (Corning). In brief, cells transfected with As-miR-10b, As-miR-222, As-miR-10b/222, and miR-Scr were seeded at a density of 5 × 10^4^ cells per upper well in 200 μL of culture medium (DMEM/F12, 4% FBS), and the lower chamber was filled with 500 μL of medium (DMEM/F12, 50% FBS). After 24 h, the upper surface was removed by scrubbing with a cotton-tipped stick, while the lower surface was fixed with methanol for five minutes, air-dried, and stained with hematoxylin and eosin. All experiments were performed in triplicate.

### Flow cytometry assays

Cells were transfected with As-miR-222, As-miR-10b, As-miR-222/10b, and miR-Scr for 48 h. The medium was replaced with serum-free medium for 24 h, and the cells were collected and fixed with 75% ethanol at 4 °C overnight. The supernatant was discarded, and the cells were washed twice with ice-cold phosphate-buffered saline. The cells were re-suspended in 500 μL propidium iodide (BD) staining buffer for 30 min at room temperature. Stained cells were analyzed on a FACSCanto II (BD Biosciences, USA). The FITC Annexin V Apoptosis Detection Kit I (BD Pharmingen;559763) was used to detect and quantify apoptosis by flow cytometry. In brief, U87MG, U251 and LN229 cells in the log phase of growth reached 70–80% confluence. After 36 h or 72 h of transfection as above mentioned, the cells were harvested and collected by centrifugation. Cells were re-suspended (1 × 10^6^ cells/ml) with binding buffer. Then added 5 μl of FITC annexin V and 5 μl PI and incubated for 15 min. Then, the stained cells were immediately analyzed using by FACSCanto II.

### RNA extraction, cDNA synthesis and quantitative real-time PCR

Total RNA was isolated from cell lines using TRIzol reagent (Invitrogen;15596026). The cDNAs were prepared with the use of PrimeScript RT reagents Kit (TaKaRa; RR037) as the manufacturer’s protocol. QRT-PCR was performed in LightCycler2.0 (Roche Diagnostics, USA) in triplicate and normalized with glyceraldehyde 3-phosphatedehydrogenase (GAPDH) or U6 as endogenous control. The real-time PCR primers were shown in Supplementary Table S8. The primers for detection of miRNA and U6 were designed by GenePharma (Suzhou, China). The primers for detection of genes were designed by Sangon Biotech (Shanghai, China).

### Immunofluorescence, immunohistochemistry assays

Immunofluorescence(IF) and immunohistochemistry(IHC) assay were performed as previously described [43, 44]. The cell lines were treated with transfections as above described. After 48 h, the cells were fixed in 4% paraformaldehyde, permeabilized with 0.1% Triton, blocked with 1 % BSA, and incubated with mouse anti-MDM2 antibody (Abcam; ab16895,1:200), rabbit anti-PTEN antibody (Proteintech;22034-1-AP,1:200) and Alexa Fluor 594 and 488-labeled secondary antibody (1:1000, Invitrogen). Nuclei were counterstained using 4′,6-diamidino-2- phenylin- dole (DAPI; Sigma; 28718-90-3). The slides were examined by fluorescence microscope (Nikon, Japan). Rabbit anti-p53(Proteintech;10442-1-AP,1:800) and mouse anti-PTEN(Proteintech;60300-1-lg,1:800) were used for IHC.

### Western blot, immunoprecipitation analysis

Western blotting and immunofluorescence assays were performed as previously described [45]. Primary antibodies included mouse anti-MDM2 (Abcam; ab16895,1:1000), rabbit anti-MDM2-phospho-S166 (Abcam; ab170880,1:1000), mouse anti-p53 (Cell Signaling Technology; #2524, 1:1000), rabbit anti-BAX (Cell Signaling Technology; #2774,1:1000), rabbit anti-BIM (Cell Signaling Technology; #2933, 1:1000), rabbit anti-p21/CDKN1A (Proteintech; 10355-1-AP,1:1000), rabbit anti-p53 (Proteintech; 10442-1-AP, 1:1000), rabbit anti-PTEN (Proteintech; 22034-1-AP, 1:1000), rabbit anti-p27/KIP1(Proteintech; 25614-1-AP, 1:1000), mouse anti-AKT-phospho-S473 (Proteintech; 66444-1-lg, 1:1000), rabbit anti-PUMA(Santa Cruz Biotechnology;sc-28226,1:1000). Following incubation in HRP labeled secondary antibody (Introvigen), protein bands were scanned with the ECL system and detected by Gel Doc 2000 (Bio-Rad). All Western blot results were confirmed from triplicate experiments. Nuclear and Cytoplasmic Protein Extraction Kit (Beyotime Biotechnology; P0028) was used to nuclear protein extracted. Co-immunoprecipitation (co-IP) assays were performed with PureProteome Protein A/G Mix Magnetic Beads (Merck Millipore) as described in the manufacturer’s protocol. Mouse anti-p53(Cell Signaling Technology; #2524,1:200) and mouse anti-MDM2(Abcam; ab16895,1:200) for the co-IP assays. After transfection of 48 h, cells were lysed using RIPA buffer (Thermo Fisher Scientific;89900) and incubated with 20 μL of protein-A/G PLUS-Agarose beads and 1 μg of the appropriate primary antibodies at 4 °C overnight. After washing three times with RIPA and the samples were analyzed through Western blotting.

### Luciferase reporter assay

Luciferase reporter assay was performed as described previously. In brief, 3′-UTR sequences and the mutant sequences of PTEN and BIM containing the putative miR-10b and miR-222 binding sites were cloned into the plasmid SV40-Luc-MCS report vectors (GeneChem, GV272). We designed three PTEN reporter vectors, namely P1 plasmid (miR-10b WT/miR-222 WT), P2 plasmid (miR-10b WT/miR-222 Mut), P3 plasmid (miR-10b Mut/miR-222 WT). The three BIM vectors designed as B1 plasmid (miR-10b WT/miR-222 WT), B2 plasmid (miR-10b WT/miR-222 Mut), B3 plasmid (miR-10b Mut/miR-222 WT; see Supplementary Fig. S5 and S8 for detailed sequences information). The next day, cells were co-transfected with P1-P3 and B1-B3 reporter plasmids and miR-10b and miR-222 mimics. 48 h after transfection, cell lysates were prepared, and luciferase reporter activity was quantified with a Dual-Luciferase Reporter Assay System (Promega).

To measure activation of the p53 response element, cells were seeded at 2 × 10^3^ cells/well in 96-well plates and allowed to settle overnight. The next day, cells were co-transfected with pGL4.38 [luc2P/p53 RE/Hygro] and pGL4.74 [hRluc/TK] vector plasmids (E692A, E365A Promega) (in a 10:1 mass ratio). 24 h after transfection, the cells were treated with miR-Scr, As-miR-10b, As-miR-222, and As-miR-10b/222 for 24 h and stimulation with doxorubicin (5 mM) for 18 h as control. Cell lysates were prepared 24 hours after treatment and luciferase reporter activity was quantified with a Dual-Luciferase Reporter Assay System (Promega).

### Tumor xenograft study

In brief, 5-week–old female BALB/c-nude mice were used for orthotropic xenograft models. The LN229 cells have been transfected with luciferase lentivirus. Then these cells were transfected with As-miR-222, As-miR-10b, As-miR-222/10b, miR-Scr, and intracranially injected into the right hemisphere of mouse (LN229-luc 3 × 10^5^ cells in 3 μl per mouse, all healthy mice were randomly divided into four groups, six mice/group). The intracranial tumors were measured as average radiance (photons/s/cm^2^/sr) by IVIS Lumina Imaging System (Xenogen) every week. At the end of the 3 weeks, the parallel groups of xenograft-bearing mice were killed. Cryosections (4 mm) were stained and used for IHC. These procedures were performed following approval by the Harbin Medical University Institutional Animal Care and Use Committee.

## Supplementary information

Supplementary materials

## Data Availability

http://bio-nefu.com/resource/jcdsa.
