# Supplementary material for: Stepwise detection and evaluation reveal miR-10b and miR-222 as a remarkable prognostic pair for glioblastoma
Source: Oncogene. 2019 Jul 9;38(33):6142–57. doi: 10.1038/s41388-019-0867-6 (PMC6756080; doi:10.1038/s41388-019-0867-6)
Supplement: Supplementary file 1 — Supplementary materials [file 41388_2019_867_MOESM1_ESM.docx]

**Stepwise detection and evaluation reveal miR-10b and miR-222 as a remarkable prognostic pair for glioblastoma**

Bo Sun^1,+^ , Xudong Zhao^2,+,*^, Jianguang Ming^1^, Xing Liu^3^, Daming Liu^1^ and Chuanlu Jiang^1,*^

^1^ Department of Neurosurgery, The Second Affiliated Hospital of Harbin Medical University, Harbin 150086, China

^2^ College of Information and Computer Engineering, Northeast Forestry University, Harbin 150040, China

^3^ Beijing Neurosurgical Institute, Beijing, 100050, China

+These authors contribute equally to this article.

*Correspondence and requests for materials should be equally addressed to Xudong Zhao (email: [zhaoxudong@nefu.edu.cn](mailto:zhaoxudong@nefu.edu.cn)) and Chuanlu Jiang (email: [jcl6688@163.com](mailto:jcl6688@163.com))

Supplementary materials including:

Supplementary Table S1;

Supplementary Table S2;

Supplementary Table S3;

Supplementary Table S4;

Supplementary Table S5;

Supplementary Table S6;

Supplementary Table S7;

Supplementary Table S8;

~~Supplementary Table S9;~~

Supplementary Figure S1;

Supplementary Figure S2;

Supplementary Figure S3;

Supplementary Figure S4;

Supplementary Figure S5;

Supplementary Figure S6;

Supplementary Figure S7;

Supplementary Figure S8.

Supplementary Figure S9

Supplementary Figure S10

**Supplementary Table S1.** Features of one-dimension with p values smaller than 0.001

| miRNA probe | Corr(Cox) | Z(Cox) | P(Cox) |
| --- | --- | --- | --- |
| hsa-miR-148a | 0.192056 | 4.606514 | 0.0001 |
| hsa-miR-221 | 0.283759 | 5.395549 | 0.0001 |
| hsa-miR-222 | 0.245557 | 6.33205 | 0.0001 |
| hsa-miR-34a | 0.18175 | 4.286961 | 0.0001 |
| hsa-miR-17-5p | -0.22319 | -3.36403 | 0.0006 |
| hsa-miR-204 | 0.120571 | 3.622066 | 0.0006 |
| hsa-miR-140 | -0.23733 | -3.19051 | 0.0008 |
| hsa-miR-340 | -0.46827 | -3.49757 | 0.0009 |
| hsa-miR-20a | -0.1768 | -3.16297 | 0.001 |

**Supplementary Table S2.** Features of two-dimension with p values smaller than 0.001

| miRNA probe | miRNA probe | Corr(Cox) | Corr(Cox) | Z(Cox) | Z(Cox) | P(Cox) | P(Cox) |
| --- | --- | --- | --- | --- | --- | --- | --- |
| hsa-miR-10b | hsa-miR-222 | 0.141237 | 0.306131 | 3.647219 | 7.178891 | 0.0004 | 0.0001 |
| hsa-miR-140 | hsa-miR-148a | -0.24495 | 0.19564 | -3.3193 | 4.717885 | 0.0004 | 0.0001 |
| hsa-miR-143 | hsa-miR-34a | -0.24517 | 0.232645 | -3.52301 | 5.206869 | 0.0004 | 0.0001 |
| hsa-miR-182 | hsa-miR-204 | -0.11859 | 0.148223 | -3.49709 | 4.28458 | 0.0004 | 0.0001 |
| hsa-miR-340 | hsa-miR-801 | -0.7523 | -0.22897 | -4.7672 | -4.04265 | 0.0001 | 0.0002 |
| hsa-miR-198 | hsa-miR-671 | 0.643254 | -0.64352 | 3.774598 | -3.92953 | 0.0002 | 0.0002 |
| hsa-miR-196a | hsa-miR-20a | 0.219051 | -0.21199 | 3.428359 | -3.66618 | 0.0007 | 0.0002 |
| hsa-miR-340 | hsa-miR-452 | -0.78113 | -0.28723 | -4.8128 | -3.62018 | 0.0001 | 0.0003 |
| hsa-miR-196a | hsa-miR-20b | 0.215901 | -0.25821 | 3.397232 | -3.61628 | 0.0008 | 0.0003 |
| hsa-miR-196a | hsa-miR-340 | 0.211458 | -0.53251 | 3.288929 | -3.8183 | 0.001 | 0.0003 |
| hsa-miR-374 | hsa-miR-671 | -0.38448 | -0.27704 | -4.18825 | -3.58374 | 0.0002 | 0.0004 |
| hsa-miR-140 | hsa-miR-801 | -0.36197 | -0.20022 | -4.27024 | -3.62355 | 0.0001 | 0.0005 |
| hsa-miR-340 | hsa-miR-671 | -0.75535 | -0.25125 | -4.66733 | -3.4952 | 0.0002 | 0.0005 |
| hsa-miR-340 | hsa-miR-765 | -0.76521 | -0.25237 | -4.67914 | -3.46795 | 0.0001 | 0.0006 |
| hsa-miR-17-5p | hsa-miR-196a | -0.2635 | 0.222578 | -3.86659 | 3.476454 | 0.0001 | 0.0006 |
| hsa-miR-222 | hsa-miR-422b | 0.291064 | -0.3619 | 7.060687 | -3.5045 | 0.0001 | 0.0007 |
| hsa-miR-140 | hsa-miR-671 | -0.39483 | -0.23331 | -4.28864 | -3.30766 | 0.0001 | 0.0007 |
| hsa-miR-340 | hsa-miR-370 | -0.78849 | -0.12011 | -4.6899 | -3.43864 | 0.0001 | 0.0007 |
| hsa-miR-374 | hsa-miR-663 | -0.3226 | -0.25508 | -3.92651 | -3.40325 | 0.0002 | 0.0007 |
| hsa-miR-190 | hsa-miR-374 | 0.947864 | -0.2649 | 3.46653 | -3.53699 | 0.0004 | 0.0007 |
| hsa-miR-148a | hsa-miR-30e-3p | 0.228711 | -0.35514 | 5.183094 | -3.19494 | 0.0001 | 0.0008 |
| hsa-miR-374 | hsa-miR-801 | -0.29315 | -0.19213 | -3.7141 | -3.439 | 0.0005 | 0.0008 |
| hsa-miR-374 | hsa-miR-765 | -0.34807 | -0.24572 | -3.94804 | -3.23458 | 0.0002 | 0.0009 |
| hsa-miR-30e-3p | hsa-miR-663 | -0.45644 | -0.25167 | -3.43876 | -3.21658 | 0.0005 | 0.0009 |
| hsa-miR-181c | hsa-miR-675 | -0.26176 | -2.92792 | -3.67551 | -3.36457 | 0.0003 | 0.001 |
| hsa-miR-200b | hsa-miR-487b | 0.454327 | 0.242446 | 4.004813 | 3.297222 | 0.0007 | 0.001 |

**Supplementary Table S3.** Features of three-dimension with p values smaller than 0.001

| miRNA probe | miRNA probe | miRNA probe | Corr(Cox) | Corr(Cox) | Corr(Cox) | Z(Cox) | Z(Cox) | Z(Cox) | P(Cox) | P(Cox) | P(Cox) |
| --- | --- | --- | --- | --- | --- | --- | --- | --- | --- | --- | --- |
| hsa-miR-106a | hsa-miR-30e-3p | hsa-miR-671 | -0.29832 | -0.47862 | -0.30979 | -4.19529 | -3.71118 | -4.08997 | 0.0001 | 0.0001 | 0.0001 |
| hsa-miR-18a | hsa-miR-30e-3p | hsa-miR-671 | -0.369 | -0.54277 | -0.30748 | -3.78283 | -4.06779 | -4.03068 | 0.0002 | 0.0001 | 0.0001 |
| hsa-miR-10b | hsa-miR-17-5p | hsa-miR-204 | 0.143551 | -0.31859 | 0.144223 | 3.585565 | -4.23067 | 4.130742 | 0.0003 | 0.0001 | 0.0001 |
| hsa-miR-143 | hsa-miR-148a | hsa-miR-221 | -0.24822 | 0.165662 | 0.265596 | -3.62028 | 3.762512 | 4.860676 | 0.0005 | 0.0001 | 0.0001 |
| hsa-miR-143 | hsa-miR-148a | hsa-miR-222 | -0.23608 | 0.173519 | 0.230714 | -3.53568 | 3.905278 | 5.871143 | 0.0005 | 0.0001 | 0.0001 |
| hsa-miR-10b | hsa-miR-204 | hsa-miR-20a | 0.137062 | 0.148701 | -0.26004 | 3.45336 | 4.272397 | -4.06615 | 0.0007 | 0.0001 | 0.0001 |
| hsa-miR-10b | hsa-miR-181c | hsa-miR-204 | 0.128182 | -0.27884 | 0.165647 | 3.292408 | -3.80423 | 4.772763 | 0.0009 | 0.0002 | 0.0001 |
| hsa-miR-17-5p | hsa-miR-30e-3p | hsa-miR-671 | -0.33657 | -0.4437 | -0.31951 | -4.45466 | -3.47064 | -4.22329 | 0.0001 | 0.0004 | 0.0001 |
| hsa-miR-182 | hsa-miR-196a | hsa-miR-204 | -0.13769 | 0.244231 | 0.15964 | -3.98737 | 3.703472 | 4.573055 | 0.0001 | 0.0004 | 0.0001 |
| hsa-miR-17-5p | hsa-miR-340 | hsa-miR-370 | -0.27195 | -0.64718 | -0.16022 | -3.33372 | -3.81435 | -4.35356 | 0.0005 | 0.0004 | 0.0001 |
| hsa-miR-10b | hsa-miR-20b | hsa-miR-221 | 0.16036 | -0.28392 | 0.321441 | 3.939011 | -3.46718 | 5.690417 | 0.0001 | 0.0005 | 0.0001 |
| hsa-miR-148a | hsa-miR-153 | hsa-miR-340 | 0.208839 | 0.959338 | -0.61773 | 4.777604 | 3.355799 | -4.22267 | 0.0001 | 0.0005 | 0.0001 |
| hsa-miR-10b | hsa-miR-20b | hsa-miR-34a | 0.133158 | -0.28515 | 0.191279 | 3.342051 | -3.52676 | 4.305157 | 0.0005 | 0.0006 | 0.0001 |
| hsa-miR-106a | hsa-miR-30e-3p | hsa-miR-370 | -0.30375 | -0.41358 | -0.13936 | -4.33666 | -3.44076 | -3.76589 | 0.0001 | 0.0007 | 0.0001 |
| hsa-miR-20a | hsa-miR-30e-3p | hsa-miR-671 | -0.26131 | -0.42602 | -0.30534 | -4.16132 | -3.33141 | -4.04349 | 0.0001 | 0.0007 | 0.0001 |
| hsa-miR-10b | hsa-miR-17-5p | hsa-miR-221 | 0.154852 | -0.26561 | 0.31441 | 3.862675 | -3.39897 | 5.502864 | 0.0002 | 0.0007 | 0.0001 |
| hsa-miR-148a | hsa-miR-190 | hsa-miR-30e-3p | 0.233494 | 0.943451 | -0.47475 | 5.274541 | 3.372486 | -3.97855 | 0.0001 | 0.0008 | 0.0001 |
| hsa-miR-17-5p | hsa-miR-30e-3p | hsa-miR-370 | -0.35876 | -0.3881 | -0.15179 | -4.68059 | -3.2444 | -4.04458 | 0.0001 | 0.0008 | 0.0001 |
| hsa-miR-29b | hsa-miR-370 | hsa-miR-374 | 0.211515 | -0.12322 | -0.41967 | 3.33676 | -3.33328 | -4.48871 | 0.0008 | 0.0008 | 0.0001 |
| hsa-miR-181c | hsa-miR-19a | hsa-miR-671 | -0.26062 | -0.25656 | -0.26175 | -3.47141 | -3.71885 | -3.70877 | 0.0005 | 0.0001 | 0.0002 |
| hsa-miR-143 | hsa-miR-148a | hsa-miR-34a | -0.27164 | 0.16865 | 0.181002 | -3.84721 | 3.698061 | 3.887227 | 0.0003 | 0.0002 | 0.0002 |
| hsa-miR-181c | hsa-miR-196a | hsa-miR-204 | -0.23504 | 0.237006 | 0.140683 | -3.39847 | 3.647641 | 4.218888 | 0.0007 | 0.0003 | 0.0002 |
| hsa-miR-148a | hsa-miR-29c | hsa-miR-30e-5p | 0.205888 | 0.303134 | -0.37855 | 4.859659 | 3.575857 | -3.70865 | 0.0001 | 0.0004 | 0.0002 |
| hsa-miR-106a | hsa-miR-30e-3p | hsa-miR-765 | -0.29343 | -0.44985 | -0.29949 | -4.20336 | -3.58709 | -3.90609 | 0.0001 | 0.0005 | 0.0002 |
| hsa-miR-30e-5p | hsa-miR-608 | hsa-miR-92 | -0.35864 | -2.86965 | -0.32387 | -3.55439 | -3.47009 | -3.93569 | 0.0002 | 0.0006 | 0.0002 |
| hsa-miR-30e-5p | hsa-miR-675 | hsa-miR-92 | -0.33059 | -3.00726 | -0.31335 | -3.38369 | -3.45146 | -3.86579 | 0.0006 | 0.0006 | 0.0002 |
| hsa-miR-198 | hsa-miR-19a | hsa-miR-671 | 0.599364 | -0.22985 | -0.67561 | 3.491745 | -3.28964 | -4.13561 | 0.0006 | 0.0008 | 0.0002 |
| hsa-miR-17-5p | hsa-miR-30e-3p | hsa-miR-765 | -0.33069 | -0.41464 | -0.3073 | -4.42994 | -3.33108 | -4.02252 | 0.0001 | 0.0009 | 0.0002 |
| hsa-miR-10b | hsa-miR-204 | hsa-miR-20b | 0.143999 | 0.143112 | -0.32643 | 3.571478 | 4.161361 | -4.05839 | 0.0003 | 0.0001 | 0.0003 |
| hsa-miR-17-3p | hsa-miR-370 | hsa-miR-374 | -0.35368 | -0.14136 | -0.33585 | -3.71252 | -3.81312 | -3.82441 | 0.0006 | 0.0001 | 0.0003 |
| hsa-miR-148a | hsa-miR-190 | hsa-miR-374 | 0.215091 | 0.990688 | -0.31599 | 5.012391 | 3.653528 | -4.08175 | 0.0001 | 0.0003 | 0.0003 |
| hsa-miR-18a | hsa-miR-30e-3p | hsa-miR-765 | -0.35655 | -0.50546 | -0.28795 | -3.69685 | -3.89533 | -3.76456 | 0.0003 | 0.0003 | 0.0003 |
| hsa-miR-190 | hsa-miR-29a | hsa-miR-30e-5p | 0.922636 | 0.288485 | -0.35569 | 3.274041 | 3.46097 | -3.51891 | 0.0008 | 0.0004 | 0.0003 |
| hsa-miR-181c | hsa-miR-19a | hsa-miR-608 | -0.26383 | -0.21473 | -3.12135 | -3.57197 | -3.36501 | -3.75396 | 0.0004 | 0.0005 | 0.0003 |
| hsa-miR-17-5p | hsa-miR-30e-3p | hsa-miR-638 | -0.29061 | -0.40873 | -0.21086 | -3.98515 | -3.18321 | -3.51681 | 0.0001 | 0.0008 | 0.0003 |
| hsa-miR-190 | hsa-miR-196a | hsa-miR-374 | 0.925714 | 0.221316 | -0.31841 | 3.402827 | 3.432127 | -4.10598 | 0.0005 | 0.0009 | 0.0003 |
| hsa-miR-148a | hsa-miR-30e-3p | hsa-miR-675 | 0.25585 | -0.55295 | -3.20934 | 5.623252 | -4.42633 | -3.75364 | 0.0001 | 0.0001 | 0.0004 |
| hsa-miR-221 | hsa-miR-374 | hsa-miR-422b | 0.325655 | -0.29282 | -0.42234 | 5.878853 | -3.67521 | -3.80331 | 0.0001 | 0.0005 | 0.0004 |
| hsa-miR-196a | hsa-miR-204 | hsa-miR-20a | 0.235043 | 0.122667 | -0.20911 | 3.622574 | 3.654785 | -3.50969 | 0.0003 | 0.0005 | 0.0004 |
| hsa-miR-18a | hsa-miR-30e-3p | hsa-miR-608 | -0.30893 | -0.4478 | -3.18132 | -3.38731 | -3.72025 | -3.78013 | 0.0007 | 0.0002 | 0.0005 |
| hsa-miR-181c | hsa-miR-19a | hsa-miR-765 | -0.24948 | -0.23938 | -0.24462 | -3.36688 | -3.58388 | -3.4197 | 0.0008 | 0.0002 | 0.0005 |
| hsa-miR-198 | hsa-miR-34a | hsa-miR-671 | 0.612902 | 0.167962 | -0.58821 | 3.480206 | 3.901544 | -3.49745 | 0.0009 | 0.0002 | 0.0005 |
| hsa-miR-30e-3p | hsa-miR-608 | hsa-miR-92 | -0.48009 | -2.9982 | -0.29001 | -3.85331 | -3.63727 | -3.59148 | 0.0002 | 0.0005 | 0.0005 |
| hsa-miR-19b | hsa-miR-30e-3p | hsa-miR-671 | -0.22482 | -0.43453 | -0.26806 | -3.36679 | -3.32319 | -3.59241 | 0.0004 | 0.0006 | 0.0005 |
| hsa-miR-140 | hsa-miR-196a | hsa-miR-31 | -0.31138 | 0.231574 | 0.270511 | -4.04702 | 3.501498 | 3.556293 | 0.0001 | 0.0007 | 0.0005 |
| hsa-miR-18a | hsa-miR-30e-3p | hsa-miR-638 | -0.31143 | -0.49526 | -0.20446 | -3.33464 | -3.71932 | -3.36892 | 0.0009 | 0.0001 | 0.0006 |
| hsa-miR-18a | hsa-miR-30e-3p | hsa-miR-370 | -0.36391 | -0.47288 | -0.13097 | -3.7655 | -3.7552 | -3.54787 | 0.0002 | 0.0002 | 0.0006 |
| hsa-miR-31 | hsa-miR-374 | hsa-miR-608 | 0.296616 | -0.31095 | -2.93409 | 3.765839 | -4.03677 | -3.56001 | 0.0004 | 0.0002 | 0.0006 |
| hsa-miR-143 | hsa-miR-181c | hsa-miR-671 | -0.23357 | -0.30317 | -0.24992 | -3.32139 | -4.09912 | -3.54265 | 0.0009 | 0.0002 | 0.0006 |
| hsa-miR-140 | hsa-miR-31 | hsa-miR-608 | -0.37271 | 0.314823 | -2.82789 | -4.50469 | 4.002222 | -3.57255 | 0.0001 | 0.0003 | 0.0006 |
| hsa-miR-140 | hsa-miR-31 | hsa-miR-675 | -0.35196 | 0.304401 | -2.93149 | -4.42011 | 3.953834 | -3.56731 | 0.0001 | 0.0003 | 0.0006 |
| hsa-miR-30e-3p | hsa-miR-675 | hsa-miR-92 | -0.46694 | -3.27354 | -0.29235 | -3.82918 | -3.75211 | -3.62619 | 0.0001 | 0.0004 | 0.0006 |
| hsa-miR-30e-3p | hsa-miR-31 | hsa-miR-675 | -0.42495 | 0.278484 | -3.1585 | -3.52502 | 3.620864 | -3.63995 | 0.0005 | 0.0005 | 0.0006 |
| hsa-miR-30e-3p | hsa-miR-31 | hsa-miR-608 | -0.44309 | 0.285955 | -2.9704 | -3.58268 | 3.632152 | -3.58262 | 0.0004 | 0.0006 | 0.0006 |
| hsa-miR-17-5p | hsa-miR-30e-3p | hsa-miR-663 | -0.24302 | -0.40742 | -0.27814 | -3.50518 | -3.15859 | -3.5954 | 0.0004 | 0.0008 | 0.0006 |
| hsa-miR-370 | hsa-miR-374 | hsa-miR-92 | -0.14196 | -0.39344 | -0.27648 | -3.77292 | -4.3477 | -3.5312 | 0.0002 | 0.0001 | 0.0007 |
| hsa-miR-106a | hsa-miR-30e-3p | hsa-miR-638 | -0.25072 | -0.4335 | -0.20091 | -3.70245 | -3.36719 | -3.36233 | 0.0004 | 0.0005 | 0.0007 |
| hsa-miR-106a | hsa-miR-30e-3p | hsa-miR-608 | -0.22662 | -0.37667 | -2.90537 | -3.52393 | -3.21183 | -3.55422 | 0.0004 | 0.0009 | 0.0007 |
| hsa-miR-148a | hsa-miR-222 | hsa-miR-223 | 0.178558 | 0.268087 | -0.1892 | 4.00661 | 6.314662 | -3.42508 | 0.0001 | 0.0001 | 0.0008 |
| hsa-miR-140 | hsa-miR-370 | hsa-miR-92 | -0.4112 | -0.11932 | -0.2685 | -4.38457 | -3.46804 | -3.44147 | 0.0001 | 0.0002 | 0.0008 |
| hsa-miR-17-5p | hsa-miR-196a | hsa-miR-204 | -0.2508 | 0.237385 | 0.11864 | -3.59494 | 3.65122 | 3.515852 | 0.0004 | 0.0003 | 0.0008 |
| hsa-miR-18a | hsa-miR-30e-3p | hsa-miR-560 | -0.30381 | -0.43744 | -0.41245 | -3.33355 | -3.53717 | -3.26294 | 0.0009 | 0.0003 | 0.0008 |
| hsa-miR-17-3p | hsa-miR-608 | hsa-miR-9 | -0.3519 | -2.89204 | -0.13016 | -3.82197 | -3.57476 | -3.14712 | 0.0001 | 0.0007 | 0.0008 |
| hsa-miR-19a | hsa-miR-370 | hsa-miR-504 | -0.28688 | -0.10763 | -0.66912 | -4.03444 | -3.26421 | -3.69191 | 0.0001 | 0.0008 | 0.0008 |
| hsa-miR-190 | hsa-miR-204 | hsa-miR-26b | 0.978958 | 0.151299 | -0.26707 | 3.3817 | 4.290425 | -3.34694 | 0.0004 | 0.0001 | 0.0009 |
| hsa-miR-374 | hsa-miR-422b | hsa-miR-504 | -0.30552 | -0.42741 | -0.69985 | -3.69614 | -3.83159 | -3.77765 | 0.0006 | 0.0003 | 0.0009 |
| hsa-miR-374 | hsa-miR-422a | hsa-miR-504 | -0.2998 | -0.91321 | -0.67718 | -3.61705 | -3.32617 | -3.6434 | 0.0005 | 0.0006 | 0.0009 |
| hsa-miR-181c | hsa-miR-196a | hsa-miR-99b | -0.29155 | 0.222839 | 0.306666 | -3.89288 | 3.467079 | 3.353881 | 0.0002 | 0.0008 | 0.0009 |

**Supplementary Table S4.** Features of two-dimension which are not only consistent with survival time but also associated with the stratification of patients with different survival risks (p<=0.001)

| miRNA probe | miRNA probe | Z(long-rank) | P(long-rank) | Corr(Cox) | Corr(Cox) | Z(Cox) | Z(Cox) | P(Cox) | P(Cox) |
| --- | --- | --- | --- | --- | --- | --- | --- | --- | --- |
| hsa-miR-10b | hsa-miR-222 | 3.945213 | 0.0002 | 0.14123729 | 0.306130851 | 3.647219 | 7.178891 | 0.0004 | 0.0001 |
| hsa-miR-196a | hsa-miR-20a | 3.739281 | 0.0003 | 0.21905091 | -0.21198814 | 3.428359 | -3.66618 | 0.0007 | 0.0002 |
| hsa-miR-222 | hsa-miR-422b | 3.720505 | 0.0003 | 0.29106411 | -0.361900103 | 7.060687 | -3.5045 | 0.0001 | 0.0007 |
| hsa-miR-140 | hsa-miR-148a | 3.490704 | 0.0007 | -0.2449508 | 0.195639773 | -3.3193 | 4.717885 | 0.0004 | 0.0001 |
| hsa-miR-196a | hsa-miR-340 | 3.466433 | 0.0007 | 0.21145829 | -0.532507608 | 3.288929 | -3.8183 | 0.001 | 0.0003 |
| hsa-miR-340 | hsa-miR-765 | 3.563095 | 0.0009 | -0.765214 | -0.252369462 | -4.67914 | -3.46795 | 0.0001 | 0.0006 |

**Supplementary Table S5**. Intersection of the pathways targeted by miR-10b and miR-222*

| **KEGG pathway** | **P-Value** | **No of predicted targets in pathway genes** |
| --- | --- | --- |
| Viral carcinogenesis | 9.41E-09 | 33 |
| Central carbon metabolism in cancer | 0.001091253 | 12 |
| **p53 signaling pathway** | **0.003038566** | **17** |
| **Cell cycle** | **0.004133863** | **22** |

*Analysis of intersection pathway was performed using DIANA miRPath, P<0.05；

**Supplementary Table S6.** The sequence of miRNA mimics, miRNA Inhibitor, miR-Scr, Inhibitor NC.

| has-miR-10b-5p  Mimics | Sense: 5’ UACCCUGUAGAACCGAAUUUGUG 3’  Antisense: 5’CAAAUUCGGUUCUACAGGGUAUU 3’ |
| --- | --- |
| has-miR-222-3p  mimics | Sense: 5’AGCUACAUCUGGCUACUGGGU 3’  Antisense: 5’CCAGUAGCCAGAUGUAGCUUU 3’ |
| has-miR-10b-5p  inhibitor | 5’CACAAAUUCGGUUCUACAGGGUA 3’ |
| has-miR-222-3p  inhibitor | 5’ACCCAGUAGCCAGAUGUAGCU 3’ |
| Inhibitor N.C | 5’CAGUACUUUUGUGUAGUACAA 3’ |
| miR-Scr | Sense: 5’UUCUCCGAACGUGUCACGUTT 3’  Antisense: 5’ACGUGACACGUUCGGAGAATT 3’ |

**Supplementary Table S7.** The sequence of shMDM2 and pLKO.1-Puro plasmid profile.

Sequence of shMDM2

sh1 CCGGCTTTGGTAGTGGAATAGTGAACTCGAGTTCACTATTCCACTACCAAAGTTTTT

sh2 CCGGGATTCCAGAGAGTCATGTGTTCTCGAGAACACATGACTCTCTGGAATCTTTTT

sh3 CCGGCGATTATATGATGAGAAGCAACTCGAGTTGCTTCTCATCATATAATCGTTTTT

sh4 CCGGCTGTGTGTAATAAGGGAGATACTCGAGTATCTCCCTTATTACACACAGTTTTT

**Supplementary Table S8.** Primer sequences used in qRT-PCR

**Primer Sequence 5’- 3**’

***BCL2L11*** rev CCAATACGCCGCAACTCT

***BCL2L11*** for ACAAACCCCAAGTCCTCCTT

***PTEN*** rev ACCAGTTCGTCCCTTTCCA

***PTEN*** for AGACCATAACCCACCACAGC

***MDM2*** rev GGAAGCCAATTCTCACGAAG

***MDM2*** for GGCAGGGGAGAGTGATACAG

***BAX*** rev GGTGAGGAGGCTTGAGGAGT

***BAX*** for GGGTTGTCGCCCTTTTCTAC

***BCL2*** rev GGAGCAGCCAGGAGAAATCAA

***BCL2*** for ATGTGTGTGGAGAGCGTCAA

***CDKN1B*** rev GGGGAACCGTCTGAAACAT

***CDKN1B*** for CTGAGGACACGCATTTGGT

***GAPDH*** for ACGACCACTTTGTCAAGCTC

***GAPDH*** rev GGTCTACATGGCAACTGAGA


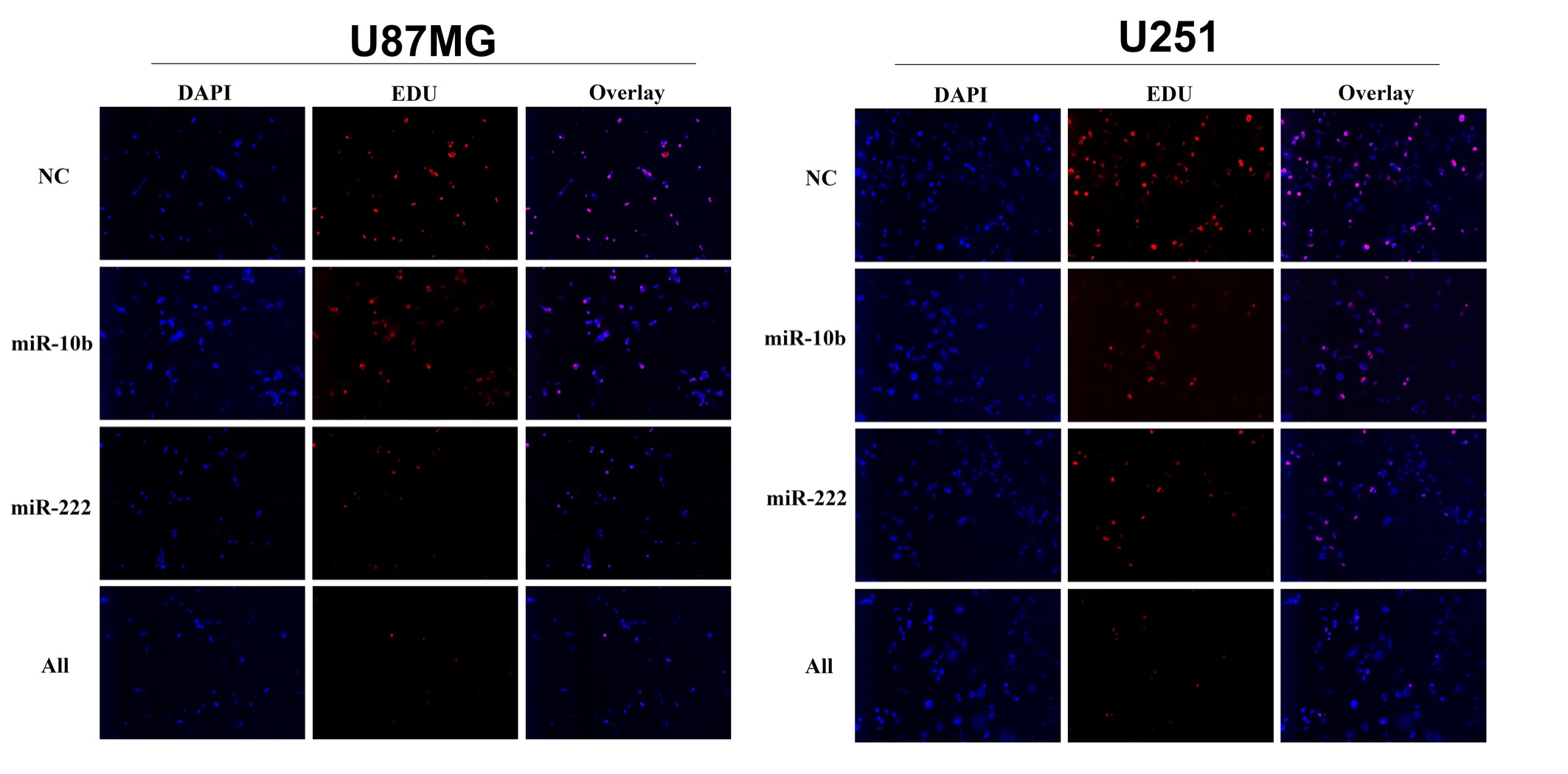


**Supplementary Figure S1.** Representative results of the EdU proliferation assay of U87MG and U251. All cells were transfected with miRNA inhibitors and labelled with EdU (red), Hoechst 33342 (blue).

**Supplementary Figure S2.** The RT-PCR results for *TP53* mRNA in LN229 and U87MG that treated with miRNA inhibitors or mimics.


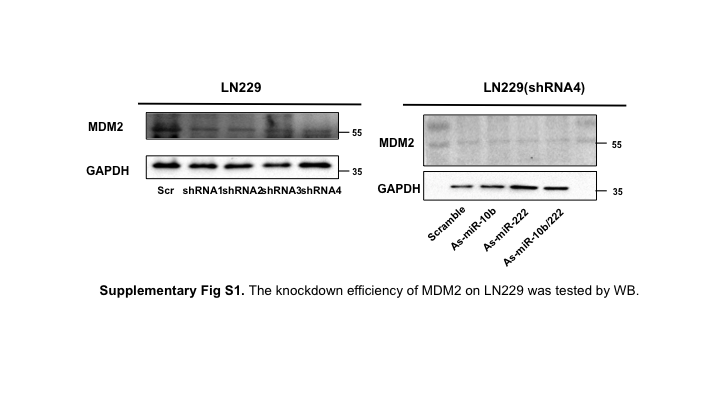


**Supplementary Fig S3.** The knockdown efficiency of MDM2 on LN229 was tested by WB.

**Supplementary Fig S4.** Co-IP assay to investigate the interaction between MDM2 and p53 among the four transfection groups.


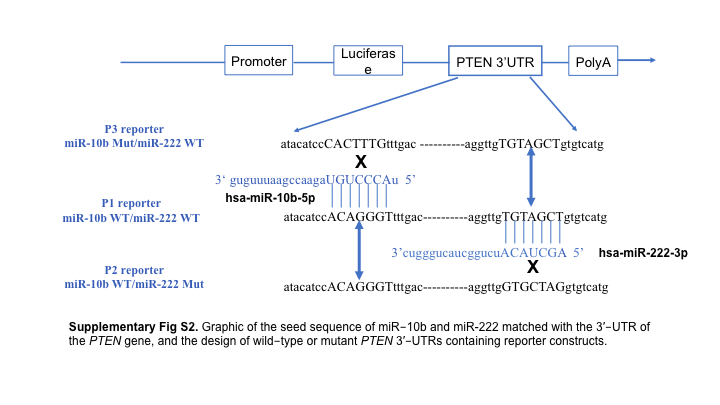


**Supplementary Fig S5.** Graphic of the seed sequence of miR-10b and miR-222 matched with the 3’-UTR of the PTEN gene, and the design of wild‐type or mutant PTEN 3’-UTRs containing reporter constructs


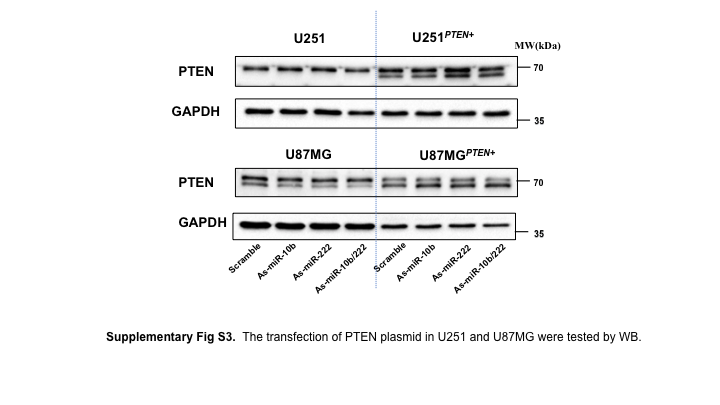


**Supplementary Fig S6.** The transfection of PTEN plasmid in U251 and U87MG were tested by WB.


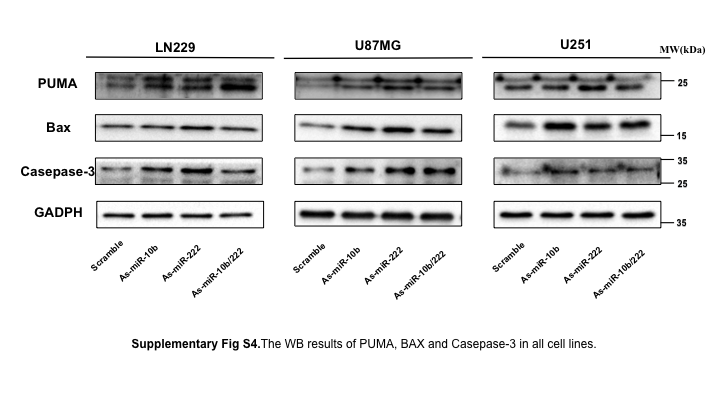


**Supplementary Fig S7.** The WB results of PUMA, BAX and Casepase-3 in all cell lines.


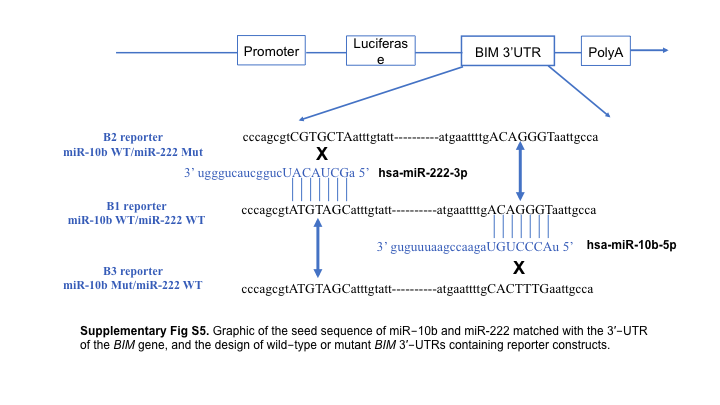


**Supplementary Fig S8.** Graphic of the seed sequence of miR-10b and miR-222 matched with the 3’-UTR of the *BIM* gene, and the design of wild-type or mutant *BIM* 3’-UTRs containing reporter constructs.


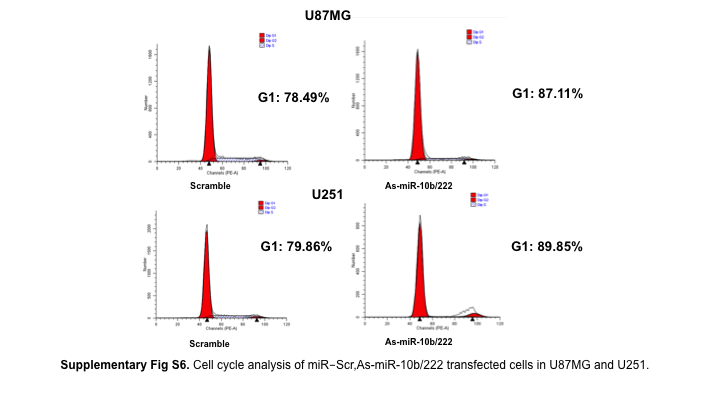


**Supplementary Fig S9.** Cell cycle analysis of miR-Scr, As-miR-10b/222 transfected cells in U87MG and U251.


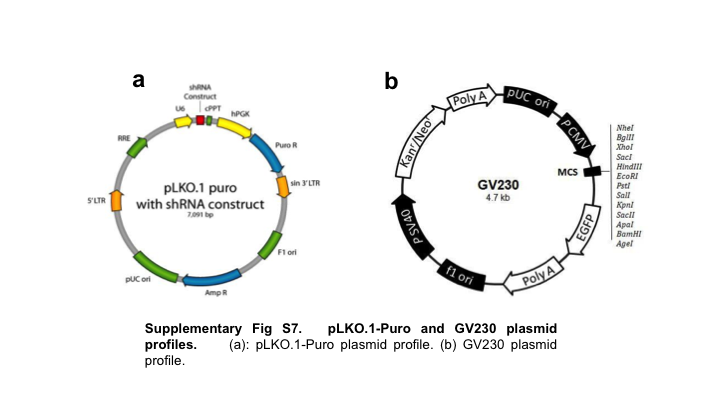


**Supplementary Fig S10. pLKO.1-Puro and GV230 plasmid profiles.** (a): pLKO.1-Puro plasmid profile. (b) GV230 plasmid profile.
